# Highlight—What You Eat: Bacterivorous Green Algae Raises Questions about the Evolution of Eating, Photosynthesis

**DOI:** 10.1093/gbe/evv208

**Published:** 2015-11-24

**Authors:** Danielle Venton

Eons ago, around 1.5 Gyr, green algae, the group that gave rise to land plants, got their start when one cell ate another cell that could harvest power from the sun. The endosymbiotic event believed to kick off one of Earth’s major eukaryotic lineages is presumed to have happened just once. And while the descending organisms retained their plastids (the site of photosynthesis) most lost their ability to “eat” or take in nutrients through phagocytosis. Such is the current understanding. But, new work from a group of researchers centered at the American Museum of Natural History suggests that the story could be more interesting than that.

“We tend to work on very unique organisms,” says John Burns, a postdoctoral fellow at the museum. “It’s interesting to study things that are way out there in evolution.”

Burns is a coauthor of a new study in *Genome Biology and Evolution* examining the genomics of an unusual green alga Burns et al. 2015. He says he might not use the term “living fossil” but “it has this characteristic way of feeding that we think all the ancestors of plants had.”

*Cymbomonas tetramitiformis* gets energy in two ways, through run-of-the-mill photosynthesis and by phagocytosis—engulfing bacteria through a tubular channel.

“This is a really interesting organism because it sits in a pivotal evolutionary position with respect to understanding how chloroplasts, or plastids more generally, have evolved,” says John Stiller, a professor at East Carolina University in the United States who studies algal genes but was not involved in the work. “I think the findings of this paper have some really interesting implications with respect to whether our current models of chloroplast evolution make sense.”

The comparative genomics done by Burns and his coauthors Amber Paasch, Apurva Narechania, and Eunsoo Kim indicated that *C. tetramitiformis*—whose genome is more than twice as large as most other green algae genomes—should still have the pathways for lipid-A and peptidoglycan metabolism.

After sequencing *C. tetramitiformis* the researchers compared the genes found in it to a number of other green algae, red algae, and glaucophytes (a small group of freshwater algae).

Interestingly, no red algae or glaucophytes are known to be phagocytic. Yet presumed homologs of genes involved in peptidoglycan synthesis were found in glaucophytes. Not genes for lipid-A synthesis though. “The relationships between three lineages of Archaeplastida are still controversial and it is not clear which of these groups emerged earliest,” says Paweł Mackiewicz, a genomicist not involved in the work at the University of Wrocław in Poland.

For Stiller, the work opens possibilities. Phagotrophy is almost certainly ancestral to green algae he says: “And it provides a mechanism for how green algae adopted a chloroplast in the first place, because they could eat cyanobacteria and then they could incorporate that as an endosymbiotic organism.” But does it go back further (as is now the dominant view)?

“If [phagotrphy] goes back to the common ancestor of the single origin of plastids,” he says, “then it’s harder to imagine how this ancestral condition managed to be maintained in one single green alga, even though every other organism from every branch of that lineage from far more ancient endosymbiosis lost it.”

Current models of plastid and eukaryotic evolution were formed without the benefit of genomic information. Perhaps, Stiller speculates, the adoption of chloroplasts may have moved horizontally across many organisms, even more than we know.
